# Editorial for the Special Issue on Soft Actuators: Design, Fabrication and Applications

**DOI:** 10.3390/mi15070912

**Published:** 2024-07-14

**Authors:** Chongjing Cao, Bo Li, Xing Gao

**Affiliations:** 1 Research Centre for Medical Robotics and Minimally Invasive Surgical Devices, Shenzhen Institute of Advanced Technology (SIAT), Chinese Academy of Sciences, Shenzhen 518055, China; 2 School of Mechanical Engineering, Xi’an Jiaotong University, Xi’an 710049, China

The topic of soft robotics combines robotics, biology, and material sciences to develop the next generation of robots that are better suited to complex uncertain natural environments and human-centered operations with strict safety requirements [1,2,3,4,5]. As one of the core components of soft robots, soft actuators have consistently been the research focus of this field over the last two decades [6,7,8,9,10]. We must acknowledge, however, that this field still faces a set of key challenges. These include achieving the more efficient/effective actuation of soft actuators through clever and elegant design; developing rapid, yet reliable, fabrication techniques to replace conventional time-consuming casting for soft actuators; and developing novel applications for these soft actuators that exhibit their true potential in real-world settings. This Special Issue provides both comprehensive reviews on the state of the art and emerging advancements that will lead the research on the next generation of soft actuators. The articles collected in this Special Issue cover broad aspects of soft robotic systems, including novel soft sensing, soft actuation, soft–rigid stiffness programming, fabrication and control strategies, and applications of soft robotic devices in, e.g., in-pipe crawling and active vibration isolations. These articles highlight the advancements in developing the upcoming generation of soft robotic technologies and laying the groundwork for versatile and robust soft robotic applications in industry and manufacturing, interacting with humans, and exploring extreme and hazardous environments.

The first article published in this Special Issue is a review by Adnan Zain et al. [11] on microbottle resonators (MBRs) for sensing applications. MBRs are stretched resonators shaped like a bottle or spheroid that are formed by introducing radius variations into the optical fiber. The high Q factor and small mode volume of MBRs make them a good choice for sensing applications, which can be environmental, chemical, or biological. Firstly, the optical properties of MBRs are introduced in terms of the resonant wavelength, free spectral range, mode volume, quality factor, and finesse. Then, seven light coupling methods of MBRs are introduced and compared. In addition, the sensing principle and sensing parameters of MBRs are discussed. Finally, the MBRs fabrication methods and their applications in sensing are introduced. It is pointed out that single-particle detection and biomedical applications may be the future development direction of MBR sensors.

The article by Nguyen et al. [12] provides a comprehensive review of biologically inspired robotic grippers. The robotic grippers have made remarkable progress in mimicking biological mechanisms, greatly improving human daily life and industrial applications. The paper encompasses three overarching themes: human-, animal-, and plant-inspired gripper concepts. Firstly, human-hand-like grippers are reviewed from two aspects: traditional rigid-link grippers (two-finger, three-finger, and multi-finger grippers) and soft grippers (gripping by actuation, controlled stiffness, and controlled adhesion). Subsequently, the art of animal-inspired grippers is developed and explored in its diverse forms through the lens of eight major taxonomic categories: clamp, suction, wrapping, dry adhesion, wet adhesion, swallow, lock, and hook. After that, the existing plant-inspired grippers are also summarized in detail. Finally, they summarize the actuators, materials, and applications of a B-I gripper. It is anticipated that these B-I grippers will play an increasingly significant role in revolutionizing the way we interact with and manipulate the world around us.

Du et al. [13] propose a tubular soft robot with a simple structure of spring-rolled dielectric elastomer (SRDE) and compliant passive bristles. The proposed robot can operate in a pipe with an inner diameter greater or less than the bristle due to the compliance of the bristle. Firstly, the nonlinear dynamic behavior of the actuator was studied experimentally. Then, the manufacturing method of the robot was introduced, and its motion performance was further studied. When the inner diameter of the pipe is less than the outer diameter of the bristles, the bristles are in a curved state, and the robot’s movement is mainly due to anisotropic friction (1.88 and 0.88 body length/second in the horizontal and vertical directions, respectively, when the inner diameter is 18 mm). In a pipe with a larger inner diameter, applying a small bending moment on the lower bristle leg helps the robot’s movement, resulting in a high robot speed (2.78 body lengths per second in a 20 mm diameter acrylic pipe). Finally, the movement of the robot under different geometric shapes is demonstrated, such as a curved pipe and flat ground.

In the fourth article, Roshanfar et al. [14] present their investigation into a pressure-regulating stiffness tendon-driven soft robot and provide a continuum mechanics model for use in adaptive stiffness applications. Firstly, a central single-chamber pneumatic and tri-tendon driven soft robot was designed and fabricated. Then, the classical Cosserat’s rod model was extended using the hyperelastic material model, and the model was expressed as a boundary value problem and solved by the shooting method. Moreover, the parameter identification problem of the relationship between the flexural stiffness and the internal pressure of a flexible robot was established, aiming at identifying the effect of pressure reinforcement. Finally, the bending stiffness of the robot under various pressures was optimized to make the theoretical deformation match the experimental results. The theoretical and experimental results agreed with each other at any pressure with a maximum error of 6.40% of the flexural length.

Nguyen et al. [15] introduce a novel gripper design, a granular-tendon gripper, aiming to achieve more universal object grasping. The gripper design features a hybrid mechanism that leverages the soft structure provided by multiple granular pouches attached to the finger skeletons, enabling soft interaction during gripping, while having the bone support to provide force. To evaluate the performance of the gripper, a series of experiments were conducted using 15 different types of objects. The results show that the proposed fixture design achieved a high success rate when grasping objects weighing less than 210 g. Granular-tendon grippers are able to effectively adapt to a variety of objects regardless of their shape and material properties.

The article by Dämmer et al. [16] describes the functional principle, design, and manufacturing of a servo pneumatic rotary actuator that is suitable for continuous rotary motion and positioning. The proposed actuator achieved rotary motion by alternately driving nine radial linear bellows actuators, which has the advantage of being easy to manufacture and enables rapid design modification. Each bellows actuator includes an elastomeric bellows structure, a guiding mechanism, and a top cap with a roller that is in contact with the inward-facing sinusoidal cam profile of the rotor. The pressurization in the pressure phase causes the corresponding bellows actuator to expand, which in turn pushes the cam profile forward. Proportional valves and rotary encoders are used to control the relationship between the bellows pressure and the rotation angle. The manufacturing prototype has a diameter of 110 mm, a width of 41 mm, and a weight of less than 500 g, and it produces 0.53 Nm of torque at 1 bar pressure, with a static positioning accuracy of 0.31° and no angular motion restrictions.

Chen et al. [17] describe the design of a pneumatic double-joint soft actuator based on fiber winding and the building of a dexterous hand with 11 degrees of freedom. Firstly, the soft actuator structure was designed according to the actuator driving principle, and the specific manufacturing process was described. Then, the bending performance of a single actuator was analyzed, including the bending angle, speed, force magnitude, etc. The feasibility and flexibility of the robot’s dexterous hand were verified via gesture recognition and a series of grasping experiments. The results show that the five-fingered dexterous hand has the characteristics of a wide grasping range, strong adaptability, and high anthropomorphism.

Di Patrizio Stanchieri’s article [18] presents the design, implementation, and characterization of a current-mode analog-front-end circuit for capacitance-to-voltage conversion. The current mode design was adopted in this scheme. The second-generation current conveyor was used as the basic block to convert the measured capacitance change into the corresponding DC voltage signal value. The circuit allows for gain and sensitivity adjustment, offset compensation and adjustment, and the ability to manage a variety of input capacitance variation ranges. For a circuit with a gain of 1000, the measurement circuit sensitivity is equal to 167.34 mV/pF, and the capacitance resolution is 5 fF. The designed circuit was used to measure the capacitance variation in a McKibben’s pneumatic muscle. The experimental results showed that the variation in the pneumatic muscle length was linearly related to the circuit output voltage. Under the condition of the step-by-step movement of the pneumatic muscle, the overall sensitivity of the system was 70 mV/mm, and the standard deviation error of the muscle length change was 0.008 mm. This indicates that the proposed circuit architecture can be effectively used with a variety of capacitive sensors and actuators for collaborative robots in industrial and medical applications.

Wang et al. [19] present a method for the simultaneous measurement of the local pulse wave velocity (PWV) in radial arteries based on the fiber Bragg grating (FBG) technique. The pulses at the wrists and at the fossa cubitalis in each radial artery were measured simultaneously using two optical fibers, each of which was inscribed with two FBG units. Based on the measurements of five male volunteers aged 19 to 21 years old, the average left radial PWV ranged from 9.44 m/s to 12.35 m/s, and the average right radial PWV ranged from 11.50 m/s to 14.83 m/s, and for each volunteer, the average right radial PWV was higher than the average left radial PWV with an obvious difference ranging from 2.27 m/s to 3.04 m/s. This method supports the dynamic analysis of local PWV and supports the analysis of local PWV features within different arteries. This method may have high clinical application potential in the future.

The research presented in Li et al. [20] comprised the design of a new semi-active vibration absorber, which was developed by stacking plasticized polyvinyl chloride (PVC) gel actuator units. The frequency response curves of the absorber demonstrated that it has a relatively wide absorption bandwidth. The vibration absorption experiments of a steel plate showed that the absorption bandwidth of a single PVC gel absorber covered the range of three natural frequencies (76.5 Hz, 95 Hz, and 124 Hz) of a rectangular steel plate in vibration attenuation. The maximum reduction percentage in the acceleration amplitude was 63%. In addition, the PVC gel’s feature of variable stiffness gave the PVC gel absorber the capability of shift frequency. Finally, a method for setting up a vibration absorption system was proposed to provide guidance for determining the number of absorbers and designing each absorber with appropriate parameters.

We hope that this Special Issue, “Soft Actuators: Design, Fabrication and Applications”, introduces the readers to several state-of-the-art research works in these rapidly expanding fields of study as well as some critical reviews on key aspects within these fields. We would like to thank each author for their contribution. Additionally, we appreciate the reviewers for providing their valuable time and effort to review the submitted manuscripts. Finally, we would like to express our gratitude to the staff of the Editorial Office of *Micromachines*, especially to Mr. Lebron Tu and Ms. Faye Zou, for their invaluable help.
